# Book Reviews

**Published:** 1896-10

**Authors:** 


### Book Reviews.

In Sickness and in Health. A Manual of Domestic Medicine and Surgery, Hygiene, Dietetics and Nursing. Dealing in a practical way with the problems relating to the maintenance of health, the prevention and treatment of disease and the most effective aid in emergencies. Edited by J. West Roosevelt, M. D., Late Physician-in-Charge of Seton Hospital for Consumptives ; Visiting Physician to Bellevue Hospital, and Attending Physician to Roosevelt Hospital, New York. Complete in one volume of over 1000 pages. Illustrated with four colored plates and numerous engravings. Full analytical index. Sold only by subscription. Price, $6.00, in cloth binding. New York : D. Appleton & Co., 72 Fifth avenue. 1896.

This book marks the first attempt to prepare a treatise on domestic medicine by scientific men at the instance of a publishing house that has acquired fame in medical literature. The others have been written by fakirs, irregular practitioners, botanical Thompsonians, red pepper or steam doctors, eclectics or general all-round advertising frauds.

The editor of this volume, Dr. J. West Roosevelt, and all his associates are physicians of science, excepting Miss Maxwell, who is a nurse of science. The first article considers the anatomy of the human body, and is written by George Waldo Crary. The next article is entitled Physiology : the vital processes in health, of which Frederic S. Lee, Ph. D., is the author.

The third article, entitled Outlines of psychology, or a study of the human mind, is by Josiah Royce, Ph. D. The next article, on physical training, is contributed by Joseph Hamblen Sears, A. B. This is followed by an article on hygiene, by Samuel Treat Armstrong, M. D. Then comes a section on surgical injuries and surgical diseases, by Alexander B. Johnston, M. D.

The seventh article, on diseases in general, is the product of a joint authorship of the editor, Dr. J. West Roosevelt, and Dr. William P. Northrup. This is followed by an article on diseases of the digestive organs, heart and lungs, by Frank W. Jackson, M. D. Next comes an article on diseases of the kidneys and urinary derangements, by J. West Roosevelt, M. D. Next in order we find an article on diseases of women and midwifery, by Samuel Waldron Lambert, M. D.

The eleventh article, entitled Nervous and mental diseases, is contributed by Frederick Peterson, M. D. This is followed by a section on medicines and treatment, by Henry A. Griffin, M. D. The final article, entitled Nursing the sick, is contributed by Miss Anna Caroline Maxwell. The managing editor is Celeste Winans Herrick, A. B.

We have given the foregoing headings of the articles and their authors that our readers may judge of the scope of the work and the ability of the contributors. In each section there is to be found just enough of the subject dealt with to give it a flavor of science without delving so deeply into the subject as to make its reading onerous. In other words, the volume is written for the popular eye rather than for the laboratory or consulting-room. It is just such a work as may properly find itself on the library shelves of every householder without doing violence to the most fastidious sense. Indeed, the information it conveys is of a nature to make it a proper book to place in the libraries of high schools for reference by teachers and advanced students.

Sadly enough, the editor, Dr. J. West Roosevelt, a most accomplished teacher, writer and physician, departed this life just before the final pages of his book issued from the press, hence is deprived of the satisfaction of enjoying a noble service well performed. The May issue of the Journal chronicled his sudden taking off by pneumonia.

We are unwilling to close this imperfect sketch of what we are convinced will prove a useful book without a word of compliment to the publishers, who deserve the thanks of the profession of medicine for rescuing from the hands of irregulars a class of literature in which fakes and fakirs have enjoyed high carnival for many years. Notice is served on them by this work that they must give up the field to scientific men.

---

Transactions of the Southern Surgical and Gynecological Association. Eighth annual meeting, held at Washington, D. C., November 12, 13 and 14, 1896. W. E. B. Davis, M. D., secretary, Birmingham, Ala. Published by the Association. Philadelphia: Wm. J. Dornan, Printer, 100 North Seventh street. 1896.

The annual volume of transactions of this association always furnishes pleasant reading. Moreover, it is one of the most instructive of the books of its class that comes to our table. Society transactions are very often dry in material and dull in their discussions; not so with this association. It is composed of live men who are advanced thinkers in their specialties, come to the meetings with something to say and say it in an interesting, instructive and succinct manner.

There is one criticism we should like to offer, and in doing so we wish to speak in an impersonal manner and not with reference to whatever may have been already published. In this period of medicine we are unable to concede the propriety of taking up the time of an aggressive association, whose moments are valuable, in the reading of lengthy biographical sketches of men who have gone to their eternal rest. In most instances biographies and eulogies of such have already been published elsewhere, and the time occupied in reading them before busy associations might be better employed in scientific work. If thought best, they may be read by title and published in the annual volume.

The editor of this book of transactions is a skilful and experienced secretary, who puts together the three days' work of his society into the best possible shape, whether judged from a scientific or mechanical standpoint.

